# Impact of community health interventions on maternal and child health indicators in the upper east region of Ghana

**DOI:** 10.1186/s12884-023-05577-7

**Published:** 2023-04-28

**Authors:** Evelyn Sakeah, Ayaga A. Bawah, Patrick O. Asuming, Cornelius Debpuur, Paul Welaga, Timothy Awine, Maria Anyorikeya, Irene Kuwolamo, Juhwan Oh, Hoon Sang Lee, Hwa-Young Lee, Inseok Lee, Myeong Seon Kim, Seoyeon Hong, John Koku Awoonor-Williams, James F. Phillips, Patrick Aboagye, Abraham R. Oduro

**Affiliations:** 1 Navrongo Health Research Centre, Ghana Health Service, Navrongo, Ghana; 2 School of Public Health, CK Tedam University of Technology and Applied Sciences, Navrongo, Ghana; 3 Regional Institute for Population Studies, University of Ghana, Legon, Accra, Ghana; 4 Business School, University of Ghana, University of Ghana, Legon, Accra Ghana; 5 Ghana Health Service, Accra, Ghana; 6 College of Medicine, Seoul National University College of Medicine, Seoul, Republic of Korea; 7 RIGHT (Research Investment for Global Health Technology) Foundation, Seoul, Republic of Korea; 8 Graduate School of Public Health and Healthcare Management, The Catholic University of Korea, Seoul, Republic of Korea; 9 KOICA Ghana Project Implementation Unit, Accra, Ghana; 10 Caleb & Company, Seoul, Korea; 11 Seoul National University College of Medicine, Seoul, Republic of Korea; 12 Mailman School of Public Health, Columbia University, New York, USA

**Keywords:** Ghana, Health systems strengthening, Community Health Planning and Services, CHPS+, Korean International Cooperation Agency (KOICA), Community-based primary health care, Maternal and child health, Universal Health Coverage

## Abstract

**Background:**

This paper reports on results of a health system strengthening implementation research initiative conducted the Upper East Region of northern Ghana. Transformative interventions to accelerate and strengthen the health delivery were implemented that included empowering community leaders and members to actively participate in health delivery, strengthening the referral systems through the provision of community transport systems, providing basic medical equipment to community clinics, and improving the skills of critical health staff through training.

**Methods:**

A mixed method design was used to evaluate the impact of the interventions. A quantitative evaluation employed a flexible research design to test the effects of various component activities of the project. To assess impact, a pre-versus–post randomized cluster survey design was used. Qualitative research was conducted with focus group data and individual in depth interviews to gauge the views of various stakeholders associated with the implementation process.

**Results:**

After intervention, significant improvements in key maternal and child health indicators such as antenatal and postnatal care coverage were observed and increases in the proportion of deliveries occurring in health facilities and assisted by skilled health personnel relative to pre-intervention conditions. There was also increased uptake of oral rehydration salts (ORS) for treatment of childhood diarrhoea, as well as marked reductions in the incidence of upper respiratory infections (URI).

**Conclusions:**

A pre-and post-evaluation of impact suggests that the programme had a strong positive impact on the functioning of primary health care. Findings are consistent with the proposition that the coverage and content of the Ghana Community-based Health Planning and Services programme was improved by program interventions and induced discernable changes in key indicators of health system performance.

## Background

The poor status of maternal and child health in low-and middle-income countries (LMICs), especially in sub-Saharan Africa (SSA), remains a major global concern [1–4]. This paper reports on results of a project, located in Ghana’s Upper East Region (UER), that represents a progression from a three decade legacy of implementation research addressed to the excess mortality problem and the need for practical solutions to health development needs, [5, 6]

Beginning in the early 1990s, mounting evidence showed that the UER was not only Ghana’s most impoverished region, it was also the most health deprived region. The absence of progress in maternal and childhood reduction indicated that the region was unlikely to achieve the Alma Ata goal of health for all by the target year 2000, not only in the UER, but in other regions of Ghana [7]. Health facilities and basic care were remote from most UER households. To contribute to national policy deliberations, the Ministry of Health (MoH) sponsored a pilot study of the Navrongo Health Research Centre (NHRC), an officially sanctioned research station of the Ghana Health Service (GHS) that is located in the UER. Pilot activity aimed to identify feasible means of solving the accessibility problem [8, 9]. When a promising set of strategies were identified, an NHRC field experiment was conducted for assessing the childhood and fertility impact of accessible community-based primary health care (CBPHC). Trial results were soon promising, prompting the GHS to commission a transfer initiative to test the replicability of Navrongo strategies in Ghana’s Volta Region [10]. Lessons emerging from this replication were used by the MoH and the GHS to develop a national CBPHC policy in 1999 that was implemented in 2000 as the Community-based Health Services and Planning (CHPS) initiative.

*Goals and objectives*. Policy pronouncements characterized CHPS as Ghana’s flagship approach to achieving “Universal Health Coverage.” [11–13] Its strategies represent an approach to expanding the provision of basic curative and preventive integrated care that improves health and reduces maternal and child mortality [14].

In the course of its initial decade of operations, monitoring showed that the national rollout of CHPS was not progressing as initially envisioned. Questions had emerged as to whether the original conceptualization of CHPS at its birth was what is being implemented, apart from its slow pace of progress. Consequently, the MoH commissioned a team to conduct national monitoring of the programme implementation and to ascertain from both national leaders and implementers at the district level their opinions about the progress of the CHPS programme leadership [15]. Results assembled by the monitoring team provided themes that could guide reform. (1) There was a lack of practical understanding of CHPS implementation among district-level managers. (2) CHPS has become a static, clinic service programme of constructing health post rather than the type of community-driven programme that was successfully tested by the Navrongo and Nkwanta research projects. (3) Managers often delayed CHPS implementation in anticipation of resources for start-up costs that they expected the central government to purvey, rather than mobilizing local resources from communities for this purpose; (4) Contrary to these expectations, there was no provision of MoH budget-lines to cover startup costs. (5) There was heavy investment in CHPS staff recruitment and training without concomitant investment in equipment. (6) Poor leadership and supervision was pervasive.

In response to these challenges, a programme of health systems implementation research was launched in 2009 in four districts of the UER that was known as the Ghana Essential Health Intervention Programme (GEHIP) [16]. Strategic interventions that addressed the key challenges identified by the Binka et al. committee but anchored on the World Health Organizations framework of health systems strengthening comprised GEHIP treatment areas [17]. Seven UER districts served as comparison areas, while two districts, where NHRC research was ongoing, were omitted from the project.

Results from the GEHIP interventions were transformative. Impact of GEHIP interventions on health systems strengthening activities reduced neonatal mortality by approximately one half [12, 18]. An important factor contributing to GEHIP mortality impact, was the its demonstration of means of accelerating the expansion of CHPS coverage in conjunction with organizing community-engaged emergency refer [19, 20].

In response to GEHIP success, the MoH and GHS requested the Korea International Cooperation Agency (KOICA) to conduct a collaborative programme of interventions in the UER termed KOICA CHPS+. Conceptualized in 2014 CHPS + aimed to find ways to support Ghana’s CHPS programme with a specific objective of revitalizing the Community Health Volunteers (CHVs) and the community-based primary health approach that CHPS originally envisioned, while incorporating robust health system strengthening approach in the process [21]. In the process, KOICA aimed to transition successful GEHIP programme that had been focused on four districts into a region of excellence for health systems strengthening in the entire UER. This involved incorporating key elements of GEHIP into KOICA’s support for CHPS strengthening, while scaling up these interventions to all 13 UER districts. In this regard, KOICA’s operational model for supporting GHS operations was not limited to providing financial resources to a locality of the country but also purveying technical support to the GHS Regional Health Administration *CHPS +* was chosen as the project title to connote the aim of improving CHPS through revitalizing the core principle of CHPS provision of community-based primary health care (CBPHC) as originally espoused at the Alma Atta Declaration [13–15, 22–26] In this regard, the CHPS + project focused on strengthening district-wide health delivery system focusing on the essential aspect of CHPS, while incorporating some of the transformative interventions identified by GEHIP. By scaling up GEHIP innovations throughout the UER, while improving GEHIP operational functioning and documenting the process of health systems strengthening, CHPS + aimed to constitute a “region of excellence,” where best practices in achieving CHPS functionality could be a resource for developing policy, training regional and district management teams, and inspiring national progress with CBPHC development.

Specific interventions implemented in CHPS + included reactivating and empowering community actors to actively participate in health delivery, strengthening the referral systems through the provision of community transport systems, and providing medical and other equipment to CHPS + compounds, subdistrict health centres and district hospitals, to facilitate effective health care delivery. In addition, skills improvement training was provided to personal at all levels, from the community to the district level, including management and leadership training to subdistrict, district and regional managers and supervisors. To motivate the CHVs to support the health delivery effort, different incentive schemes were implemented. The overall aim of these interventions was to ensure improvement in certain health outcomes, including increased visitation of community workers known as “Community Health Officers” ( CHOs) and “Community Health Volunteers (CHVs) to households within the community to provide care, improvements in antenatal care (ANC) visits by pregnant women and mothers, increased skill deliveries, improvements in immunization, etc.

The CHPS + initiative was pursued in conjunction with a parallel CHPS + initiative, funded by the Doris Duke Charitable Foundation. The goals and interventions of this project were intended to match the KOICA CHPS + initiative, but its strategy focused on transfer of GEHIP interventions to two Volta Region Districts and two Northern Region Districts. This represented an attempt to foster scale-up of GEHIP as a supplement to the KOICA “region of excellence” paradigm [27]. *The Ghana and UER mortality context.* While the global maternal mortality ratio declined by 38 per cent from 2000 to 2017 – from 342 deaths to 211 deaths per 100,000 live births, it was less than half the 6.4 per cent annual rate needed to achieve the Sustainable Development global goal of 70 maternal deaths per 100,000 live births [2]. Globally the under-five mortality rate has dropped by 41%, from 87 deaths per 1,000 live births in 1990 to 51 per 1,000 in 2011, but the annual rate of reduction could not achieve the millennium development goal 4 (MDG 4) by 2015 in many LMICs [3]. Despite improvements in immunization rates, widespread efforts to prevent maternal-to-child transmission of HIV (PMTCT), and other initiatives that have radically increased survival among children under 5, the proportion of deaths that occur within the first month of life (the neonatal period) remained high, accounting for about 33% of overall childhood mortality. Globally, reduction in neonatal mortality rates have been significantly slower (1.8% per year) than declines in under-five mortality (2.5% per year) [28, 29].

Moreover, Ghana failed to meet the Millennium Development Goal (MDG) 4 target of reducing by two-thirds, between 1990 and 2015, the under-five mortality rate (U5MR) [30]. Despite the fact that programs have become more effective in addressing under-five mortality, the proportion of deaths occurring in the neonatal period (first 28 days after delivery) have declined marginally in recent years. The neonatal deaths (deaths occurring during the first month of life per 1000 live births) constitute about 71 per cent of infant deaths and 48 per cent of deaths in children under 5 years of age in Ghana [31]. Neonatal mortality rates (NMRs) have not improved much in the past 10 years. Between 2008 and 2014, NMR declined marginally from 30 to 1000 live births in 2008 to 29 per 1000 live births in 2014 [7, 8]. Also, in the UER, the NMR *increased* from 17 to 1000 live births in 2008 to 24 per 1000 live births in 2014 [31, 32]. Clearly, levels and trends in UER mortality comprise a challenging context for KOICA CHPS + project goals to achieve significant mortality results.

**Fig. 1:**
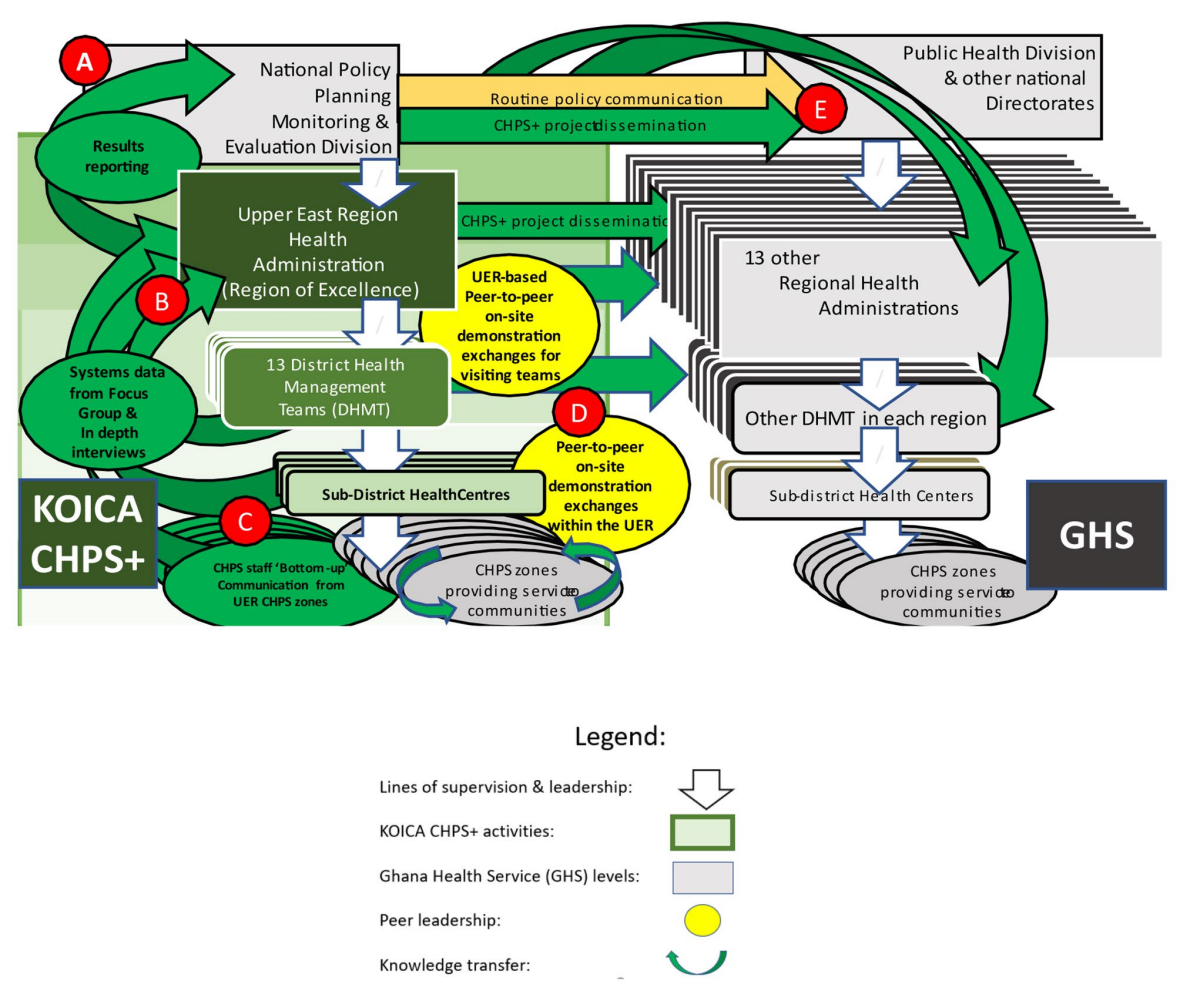
Theory of Change

*Theory of change.* The logic model articulated by KOICA CHPS + is comprised of a series of complementary interventions that aim to improve maternal and child health outcomes and survival. The project aims to achieve this result, not only in the UER, but also to impact on national program functioning. As such, it functions as an “embedded research” operation of the GHS that reports to the National Policy Planning Monitoring and Evaluation Division and functions as an activity of the UER Regional Health Administration (Fig. 1, item A). A systems strengthening approach is envisioned that is grounded in the UER but designed transfer knowledge to other regions (Fig. 1, item B). To achieve this, interventions were designed and implemented to strengthen the community component of the healthcare continuum by strengthening community volunteers through training and provision of logistics to promote healthcare, strengthening the capacity of the community health nurses through quality improvement training and provision of critical quality equipment (medical and non-medical) to the CHPS compounds and higher level health facilities such as health centres and district hospitals at the subdistrict and district levels, to facilitate delivery of quality health delivery. To strengthen overall supervision and governance in leadership and management of the district health system, leadership and management training was conducted for District Health Management Teams (DHMT) and the Regional Health Management Team (RHMT), with knowledge gained in the process absorbed by the UER RHRMT and communicated to PPME and other directorates of the GHS in Accra. The health information system was also strengthened by designing data collection tools that facilitated meticulous implementation data collection for monitoring progress of implementation. Finally, to strengthen the referral system three-wheeler motorized bikes known as “*Motorkings*” were procured and stationed in the communities to serve as emergency transport for transporting patients (women and children) to the nearest higher-level facilities. The premise is that if the manpower needs and necessary medical/non-medical equipment are provided, coupled with proper training of the cadre of health workers (community health nurses and community health volunteers) as well as sub-district and district leadership to enhance supervision, the result will be improved service delivery which will then translate into improved health and survival of mothers and children. At the community, sub-district, and district levels focus group and qualitative monitoring is applied continuously to capture information about the acceptability of operations, stakeholder views on ways to utilize results, and advice on the operational feasibility of sustaining community participation and operational effectiveness (Fig. 1, item C). Participatory observation, community and district exchanges, and peer leadership mechanisms are used to amplify the learning process (Fig. 1, item D). The different regimen of services and training are monitored in the theory of change are communication ways that permit the transfer of learning from the UER to the GHS systems, not only as project documentation, but as institutional learning that is embedded in national policy communication mechanisms (Fig. 1, item E). Taken as a systems approach to generating knowledge, communicating results, and embedding learning into operations, CHPS + institutionalizes innovation.

### Design and methods of analysis

The CHPS + project included a project evaluation system designed to (1) provide rigorous quantitative estimates of the project on desired outcomes; (2) provide detailed qualitative evidence that augments the main quantitative impact of the project; and (3) provide detailed explanation of the processes that led to quantitative impact. Accordingly, the evaluation used a mixed methods research design combining both qualitative and quantitative designs. Qualitative evaluation using in-depth interviews (IDIs) and focus group discussions (FGDs), describes the processes and procedures that explain the causal impact of the project on desired outcomes. The qualitative aspects focused on understanding knowledge acquisition, competencies, behavioral and attitudinal changes that occurred among service providers and recipients that may have led to improvement in health outcomes. The quantitative impact evaluation used survey data and other available service delivery and administrative data to provide causal estimates of the impact of the project.

The CHPS + project was composed of different interventions targeted at different levels of the healthcare system. At the community level, a total of 120 CHPS zones were randomly selected across all the districts in the Upper East region to support health service delivery at the community level using different incentive schemes as motivations, and the provision of a sustainable emergency referral system to transport mothers and children to higher level facilities. For the purpose of evaluating differential impacts of the different incentive schemes and the emergency referral system, the 120 CHPS zones were further subdivided into different sub-categories to reflect the different incentive schemes. The different interventions sought to evaluate: (1) Effectiveness of the different incentive schemes for Community Health Volunteers (CHVs); (2) Effectiveness of the Sustainable Emergency Reference System (SERC), and (3) the combined effect of the CHVs and SERC. Evaluation of CHVs incentives and SERC was based on a cluster-based randomized controlled trial, with a pre-and-post intervention data collection scheme. In addition to the community level interventions, the project also implemented interventions at the health centre (HC) level, sub-district, district and at the regional levels where personnel at the levels were provided with different types of skills through targeted training, including emergency management, leadership and management skills. The facilities at the different levels – health centres through to the regional level were provided with equipment and personnel trained on how to manage these equipment.

The quantitative evaluation employed a flexible research design that permitted testing the effects of various component activities of the project. Since many of the component interventions are at the community level and impact is measured at the individual level, the design was based on a cluster-based quasi-randomized design whereby individual-level data was collected using a well-structured survey instrument. The overall impact of the CHPS + project is based on a pre-and-post design.

To aid explanation of the quantitative results qualitative interviews were conducted at baseline and endline to clarify how the CHPS programme works, roles and activities of CHO/CHN/midwives, supervision, community engagement in the CHPS + programme and suggest appropriate ways to promote maternal and child health programmes.

While we have discussed the detailed design of the KOICA CHPS + project, it is important to note that this paper is not seeking to evaluate the different intervention subcomponents of the project; instead, we seek to report on the overall impact of the intervention on maternal health indicators pre-and-post the interventions.

### Data

The main source of data for the overall impact evaluation of the CHPS + project is two rounds of household surveys. The first round is the GEHIP end line survey which was conducted in 2014/2015. This served as the baseline data for CHPS + since KOICA’s project built on the GEHIP project. This survey interviewed more than 7600 women in their reproductive age (15–49 years) across the region on various indicators, including all the outcome indicators for the CHPS + project (see Sect. 2.2 below). The second round is an endline survey, also similar to the CHPS + midline and baseline surveys.

### Sampling

The samples for both baseline and end line quantitative surveys were designed to obtain random samples of women in their reproductive ages (15–49) that is representative of the region’s rural population. In both cases, a two-stage cluster random sampling procedure was used. In the first stage a random sample of census Enumeration Areas (EAs) was selected from the region’s rural population. To ensure proportionality according to population size, in both baseline and end-line, the number of EAs sampled from each district was based on the district’s contribution to the region’s rural population. A complete listing of all households in these EAs was conducted and this served as the sampling frame for the second stage. At the second stage sampling, a roughly equal number of households were randomly selected from the listed EAs. All eligible women (women aged 15–49 years) in the sampled households were interviewed. Households were then sampled from these strata and all women within sampled households were interviewed.

It is important to highlight some differences between the baseline and end-line sampling. First, the number of EAs in the baseline was increased from 72 to 160 EAs. The average number of women interviewed per EA decreased from baseline to end line. In both cases, sampling weights were computed and applied to account for the unequal probability that a woman is sampled across the various EAs.

It is also important to note that the data collection processes were similar across the two surveys. In both cases, electronic-based data capture using the data entry Apps and Servers of survey CTO were employed. Data collection in the baseline took place from October 2014 to February 2015 while the data collection for the end line survey took place from June-August 2020.

The analyses of the household survey were supplemented with findings from the end-line qualitative assessments of possible changes that may have been observed. The sampling for the qualitative assessments involved a multi-stage sampling method to select the respondents for the qualitative interviews. The first stage involved the selection of districts. The region was divided into three zones: east, central and west zones. In the East zone we randomly selected four districts (Bawku Municipal, Binduri, Pusiga and Garu-Tempane districts), in the central zone, we randomly selected two districts (Bongo and Talensi districts) and in the western zone we selected all four districts (Kassena-Nankana Municipal, Kassena-Nankana West, Builsa North and South Districts). In all ten districts were selected: four each from the Eastern and Western zones, and two from the Central zone. The second stage involved selecting respondents from the intervention and comparison communities. Purposive sampling method was employed to select health professionals (District Directors of Health Services and the Sub-District Heads and District Public Health Nurses, Community Health Nurses and Midwives) and community stakeholders (traditional leaders, traditional birth attendants, community health volunteers, older men and women, women and men of reproductive age) for the qualitative interviews. We identified the participants for FGDs through key informants in the communities. The FGDs and IDIs explored community stakeholders’ views about CHPS.

### Ethical approval

Ethical approvalfor the study was obtained from the Institutional Review Board of the Navrongo Health Research Centre (NHRCIRB262). All participants interviewed willingly agreed to participate in the surveys and provided informed consent.

### Limitations

A limitation of this study is the fact there may have been other programmes that may have been independently implemented by other donors that we may not be aware of which could have impacted on the results. To our knowledge however, no such large-scale interventions were concurrently ongoing at the time of the KOICA interventions. Another limitation is that the pre-post approach makes it impossible to separate the impact of the project from the expected improvements in the project’s outcome indicators overtime. Thus, the results presented in this paper could potentially overstate the impact of the project.

## Results

### Background characteristics of respondents

Table 1 reports on the background characteristics of women and children in the household surveys at two survey rounds. The total number of women surveyed at baseline was 7,693, and 4,694 at endline. The corresponding number of children was 3,501 at baseline and 2,669 at end-line.

The distribution of women interviewed showed that the sample was made of young women across the two samples (26.0% at baseline and 23.9% at endline were less than 20 years old). More than half of the women were married/cohabiting across all the two periods (60.6% at baseline and 59.0% at endline). An appreciable fraction of the women across all survey years had no formal education (48.6%, and 38.2% respectively) and only a small proportion had at least secondary or higher education, with 11.7%, and 15.5%, at baseline and endline respectively. However, the level of education improved over the two surveys. More than half of the women were illiterate at baseline (59.5%) and endline (51.7%).

The largest ethnic group was the Frafra group representing 28.0% of women at baseline and 33.9% at endline. The most dominant religion was Christianity from baseline (66.3%) to endline (69.9%). Farming and being a student was the most featured occupation of the women at baseline (27.1% and 25.8%) and endline (35.8%and 21.4%), respectively. Ownership of mobile phones increased significantly from baseline (40.8%) to endline (65.2%).

More than half of women at baseline (57.6%) had no insurance. However, the situation improved somewhat, with above half (52.4%) at endline insured. There was no clear trend in the distribution of women in the wealth groupings across the study arms even though some marginal differences were observed.

**Table 1:** Respondents Background Characteristics

INDICATOR	BASELINE (%)	ENDLINE (%)
**Number of women (N)**	7,693	4,694
**Number of Children (N)**	3,501	2,669
**Woman’s Age group**		
15–19 years	26.0	23.9
20–24 years	16.9	18.4
25–29 years	13.3	15
30–34 years	12.3	11.4
35–39 years	12.9	11
40–44 years	10.7	10.4
45–49 years	8.8	9.9
**Marital Status**		
Never Married	32.8	32.7
Currently married/cohabiting	60.6	59
Widowed	1.1	5.4
Divorced/Separated	5.6	2.9
**Education**		
None	48.6	38.2
Primary	16.1	29.4
JHS/JSS	23.6	16.9
Secondary +	11.7	15.5
**Literacy**		
Literate	40.5	48.3
Not literate	59.5	51.7
**Ethnic group**		
Buli	13.3	10.1
Kusasi	19.7	24.5
Frafra	28.0	33.9
Kassem/Nankam	22.6	13.1
Other	16.5	18.4
**Religion**		
Christianity	66.3	69.9
Traditional African Religion	8.4	4.6
Islam	23.1	24.3
No religion/Other	2.2	1.1
**Occupation**		
No Occupation	11.2	8.5
Farming	27.1	35.8
Trading/Selling	16.7	13.0
Hairdressing/dressmaking	10.0	12.4
Student	25.8	21.4
Other	9.3	9.0
**Access and or, ownership of Mobile Phone**		
Owns Phone	40.8	65.2
Has access within the compound	38.6	33.7
Access in community/no access	20.6	1.1
**Insurance Status**		
Currently insured with NHIS	42.4	52.4
Not currently insured	57.6	47.6
**Household wealth**		
Poorest	19.1	20
Poorer	19.4	20
Middle	20.4	20.4
Richer	19.9	20.9
Richest	21.1	18.7
**District**		
Bolgatanga Municipal	7.1	7.4
Bongo	9	9.7
Builsa	13.8	10.6
Kassena-Nankana Municipal	13.8	7.7
Kassena-Nankana West	9.4	7.4
Garu Tempane	17.5	15
Bawku West	7.7	9.7
Talensi-Nabdam	12	14.3
Bawku Municipal	9.6	18.1

### Impact of Project on CHPS

Table 2 presents regression results showing the effect of the KOICA CHPS + project on functioning of CHPS in the Upper East Region. The main indicators used are being visited at home by any health personnel in the last three months, being visited at home by a CHO in the last three months, being visited at home by a CHV in the last three months, and women visiting a health facility in the last three months. For each outcome we present odds-ratios from a logistic regression along with 95% confidence intervals. In each case, we present the before-after comparison between baseline and endline.

The first three columns show significant improvement in CHPS functioning between baseline and endline. The table shows statistically significant improvement in odds of being visited at home by a health personnel in the past three months (1.44 times more between baseline and endline), the odds of being visited at home by a CHO was 1.74 times more and the odds of being visited at home by a CHV was 3.34 times more, all statistically significant at 1% level. Column 4 shows there was no significant change in the likelihood of visiting a health facility in the last three months. Even though the odds-ratio shows there was a 5% reduction in the likelihood between baseline and endline, this is not statistically significant. Improvements in home visits as shown by these results have been corroborated by the qualitative findings, as recounted in the text below by respondents from in-depth interviews:

*……“ Since my wife gave birth on 13th April 2020, the nurses have been coming to her, especially when the baby is having a problem” ***(IDI_CHMC_Jagsa_Gwedema_Builsa South)**.

*….“ Community volunteers do home visits and support in immunization against certain diseases. They also mobilize community members for health activities such as health meetings, communal labour, durbars etc.” ***(FGD_Pregnant_Woman-Bok Sapiliga_Binduri)**.

Table 2 also shows that the significant predictors of women being visited at home by health personnel were age and occupation. Across the first three columns, the likelihood of being visited at home increases with age up until age 34 and thereafter records a progressive decline. Women in all occupation groups were more likely to report being visited at home compared with those with no occupation.

Column 4 of Table 2 shows that the significant predictors of visiting a health facility in the last three months are age, education, marital status, and insurance coverage. There is a non-linear relationship between age and likelihood of visiting a health facility. While women aged 20–39 were more likely to visit a health facility, those aged 40–49 were less likely to visit a health facility compared with those under 20 years. In terms of education, those with some level of education were more likely to visit a health facility compared with those without any formal education. Women who are currently or have been previously married were less likely to visit a health facility in the last one month compared with those who are never married. Finally, consistent with expectation, women who had insurance coverage were twice as likely to visit a health facility in the last three months compared with women without insurance.

**Table 2:** Effect of KOICA CHPS + project on functioning of CHPS

Covariates:	Visited at home by any health personnel in last 3 months		Visited at home by a CHO in the last 3 months		Visited at home by a CHV in last 3 months		Visited a health facility in last 3 months		
Column:	(1)		(2)		(3)		(4)		
	ORs	95% CI	ORs	95% CI	ORs	95% CI	ORs		95% CI
**CHPS + effect**: Baseline (ref)									
End line	1.44***	(1.31–1.58)	1.74***	(1.56–1.94)	3.34***	(2.82–3.97)	0.95		(0.88–1.03)
**Five year maternal age group**: 15–19 (ref)									
Ages 20–24	1.56***	(1.31–1.87)	1.27**	(1.05–1.53)	1.24*	(0.99–1.57)	2.33***	(1.93–2.81)	
Ages 25–29	1.65***	(1.33–2.04)	1.40***	(1.12–1.75)	1.14	(0.84–1.54)	3.24***	(2.53–4.14)	
Ages 30–34	1.77***	(1.39–2.26)	1.54***	(1.16–2.04)	1.30	(0.94–1.81)	3.09***	(2.44–3.91)	
Ages 35–39	1.65***	(1.29–2.09)	1.33**	(1.00–1.75)	1.25	(0.89–1.76)	2.76***	(2.13–3.59)	
Ages 40–44	1.48***	(1.14–1.92)	1.30*	(0.97–1.74)	1.29	(0.92–1.81)	2.11***	(1.61–2.77)	
Ages 44–49	1.23	(0.94–1.62)	1.07	(0.77–1.48)	1.16	(0.81–1.68)	1.80***	(1.36–2.40)	
**Maternal educational attainment**:									
None (ref)									
Primary	1.32***	(1.12–1.56)	1.14	(0.96–1.35)	1.16	(0.94–1.44)	1.42***	(1.19–1.68)	
Middle/JHS	0.97	(0.82–1.15)	0.92	(0.75–1.13)	0.76**	(0.58–1.00)	1.14	(0.96–1.36)	
Secondary	0.97	(0.79–1.19)	1.04	(0.84–1.29)	0.89	(0.69–1.14)	0.86	(0.72–1.03)	
**Household relative economic status**:									
Poorest (ref)									
Poorer	1.08	(0.91–1.28)	1.02	(0.84–1.24)	0.98	(0.77–1.24)	1.04	(0.90–1.21)	
Middle	1.06	(0.87–1.29)	1.10	(0.90–1.34)	1.01	(0.80–1.29)	1.06	(0.91–1.24)	
Wealthier	1.11	(0.93–1.33)	1.15	(0.95–1.39)	0.94	(0.74–1.21)	0.87*	(0.75–1.01)	
Most wealthy	1.04	(0.85–1.28)	1.00	(0.81–1.25)	0.94	(0.73–1.21)	0.72***	(0.61–0.85)	
**Maternal occupation**: None (ref)									
Farming	1.32***	(1.07–1.64)	1.28**	(1.01–1.63)	1.28*	(0.96–1.71)	1.54***	(1.29–1.85)	
Trading	1.32***	(1.08–1.62)	1.23*	(0.99–1.52)	1.42**	(1.05–1.91)	1.26**	(1.04–1.52)	
Hairdressing	1.25**	(1.00–1.56)	1.28**	(1.03–1.59)	1.33*	(0.97–1.81)	1.25**	(1.03–1.51)	
Student	0.93	(0.69–1.24)	1.07	(0.81–1.42)	1.45**	(1.03–2.03)	0.58***	(0.46–0.73)	
Other	1.16	(0.92–1.46)	1.17	(0.93–1.47)	1.24	(0.92–1.67)	1.35**	(1.06–1.71)	
**Maternal marital status**: Never married (ref)									
Married	2.36***	(1.92–2.90)	1.44***	(1.11–1.87)	1.18	(0.90–1.56)	4.64***	(3.83–5.61)	
Widowed	1.73***	(1.30–2.32)	1.26	(0.91–1.74)	1.15	(0.79–1.68)	2.62***	(2.00–3.43)	
Divorced	1.34	(0.93–1.92)	0.88	(0.58–1.33)	1.21	(0.79–1.88)	2.89***	(2.05–4.06)	
**Ethnicity**: Buili (ref.)									
Kusasi	0.88	(0.62–1.26)	0.76	(0.53–1.11)	0.58**	(0.38–0.88)	1.37***	(1.08–1.73)	
Frafra	1.46**	(1.08–1.97)	1.18	(0.86–1.63)	1.44*	(1.00–2.09)	1.79***	(1.40–2.30)	
Kassem	1.13	(0.82–1.57)	0.61***	(0.42–0.89)	0.86	(0.56–1.33)	0.95	(0.73–1.24)	
Ethnicity: Other	0.90	(0.63–1.27)	0.79	(0.55–1.12)	0.81	(0.55–1.19)	1.71***	(1.33–2.20)	
**Religion**: Christian (ref)									
Traditional	1.26**	(1.03–1.54)	1.26*	(0.97–1.65)	1.09	(0.82–1.45)	1.00	(0.82–1.22)	
Muslim	1.04	(0.86–1.24)	1.24**	(1.02–1.50)	1.08	(0.84–1.38)	0.99	(0.85–1.15)	
Other	1.03	(0.69–1.54)	1.05	(0.66–1.67)	1.04	(0.55–1.99)	1.05	(0.76–1.46)	
**Household health insurance status**: Not insured (ref)									
Insured	1.10*	(0.99–1.22)	1.09*	(0.99–1.21)	1.00	(0.87–1.15)	2.01***	(1.80–2.26)	
Constant	0.04***	(0.03–0.07)	0.06***	(0.04–0.09)	0.02***	(0.01–0.04)	0.10***	(0.07–0.15)	
**Summary statistics**									
**Observations**	12,386		12,386		12,386		12,386		
**Wald chi-square**	477.66		373.51		497.18		1332.95		
**Pseudo-R** ^**2**^	0.0810		0.0756		0.1868		0.2514		

Notes: Table reports results from logistic regression models. ORs denotes Odds ratio from Logistic regression. 95% CI denotes 95% confidence interval. In all regressions standard errors are clustered at enumerator area level. ***, ** and * denote statistical significance at 1%, 5% and 10% respectively

Table 3 presents the impact of the project on ANC visits, deliveries supervised by skilled health personnel and deliveries in health facilities. Column 1 presents the results for four or more ANC visits. The results show a statistically significant increase in the likelihood of four or more ANC visits by pregnant women by 76% between baseline and end line. The results also show that there was a significant positive impact on delivery supervised by skilled personnel and deliveries taking place in health facilities.

Again, results from the qualitative interviews with women in the communities show that community members themselves have noticed improvements in services provided by the health workers, as recounted by a woman in a focus group discussion session in one of the communities:

*“We have received many services from the health workers. At first when you get pregnant and no one comes to check on you then you would give birth to an unhealthy baby, but today we have the nurses weighing us and following upon us to ensure we are healthy with our unborn children*.” **(FGD-NURSING MOTHERS-NYARIGA-BONGO)**.

Column 1 of Table 3 shows that major determinants of ANC attendance are occupation, marital status and insurance coverage. The likelihood of 4 or more ANC is higher among all occupation groups compared with women with no occupation. However, the effect was statistically significant for those engaged in trading or hairdressing. Even though insurance is not required to access ANC services for pregnant women, the results still show that women with insurance coverage are more likely to have 4 or more ANC visits.

Columns 2 and 3 show that age, education, occupation, religion and insurance coverage are significant determinants of delivery in health facility and delivery supervised by skilled health personnel. Older mothers were less likely to deliver in a health facility or had a supervised skilled delivery compared with those aged 15–19 years. Skilled delivery and facility delivery are both increasing the level of education. Again, women in all occupational categories were more likely to deliver and a health facility and have their delivery supervised by skilled personnel compared with those without occupation. Finally, women with insurance were more likely to deliver in health facilities and have their deliveries supervised by health personnel.

**Table 3:** Effect of CHPS + on ANC visits and Facility Delivery

	(1)		(2)		(3)	
	Had 4 or more ANC visits		Delivered by skilled health personnel		Delivered in a health facility	
VARIABLES	ORs	95% CI	ORs	95% CI	ORs	95% CI
Baseline (ref)						
End line	1.76***	(1.53–2.02)	1.70***	(1.47–1.96)	1.70***	(1.45–1.98)
Age: 15–19 (ref)						
Age: 20–24 years	0.86	(0.48–1.54)	0.70	(0.42–1.18)	0.63*	(0.38–1.04)
Age: 25–29 years	1.03	(0.54–1.95)	0.70	(0.43–1.15)	0.57**	(0.35–0.92)
Age: 30–34 years	0.89	(0.46–1.73)	0.62*	(0.37–1.03)	0.52**	(0.32–0.87)
Age: 35–39 years	0.87	(0.44–1.70)	0.46***	(0.27–0.78)	0.43***	(0.26–0.71)
Age: 40–44 years	1.14	(0.56–2.32)	0.50***	(0.30–0.84)	0.42***	(0.25–0.71)
Age: 44–49 years	1.46	(0.55–3.85)	0.58*	(0.31–1.06)	0.47**	(0.26–0.84)
Education: none (ref)						
Education: primary	1.08	(0.76–1.53)	1.00	(0.80–1.25)	1.09	(0.86–1.38)
Education: Middle/JHS	0.96	(0.64–1.44)	1.66***	(1.18–2.33)	1.66***	(1.22–2.28)
Education: secondary+	1.56	(0.88–2.77)	3.08***	(1.83–5.20)	2.77***	(1.72–4.48)
Wealth: poorest (ref)						
Wealth: Poorer	1.14	(0.77–1.69)	1.12	(0.86–1.47)	1.05	(0.81–1.37)
Wealth: Middle	1.34*	(0.96–1.85)	1.11	(0.86–1.44)	1.10	(0.84–1.43)
Wealth: Richer	1.02	(0.73–1.43)	1.18	(0.88–1.57)	1.18	(0.89–1.56)
Wealth: Richest	1.33	(0.87–2.06)	1.24	(0.89–1.73)	1.04	(0.74–1.46)
Occupation: none (ref)						
Occupation: Farming	1.02	(0.66–1.58)	1.01	(0.76–1.34)	1.05	(0.80–1.38)
Occupation: Trading	1.69**	(1.04–2.75)	1.83***	(1.34–2.50)	1.96***	(1.44–2.67)
Occupation: hairdressing	1.62*	(0.94–2.78)	1.81***	(1.27–2.57)	1.69***	(1.20–2.38)
Occupation: Student	1.78	(0.63–5.01)	1.62	(0.66–3.94)	1.70	(0.65–4.46)
Occupation: Other	1.23	(0.66–2.31)	1.45*	(0.94–2.23)	1.82***	(1.17–2.83)
Marital: Never married						
Marital: married	1.80**	(1.11–2.91)	1.10	(0.73–1.65)	0.95	(0.63–1.42)
Marital: Widowed	5.47	(0.70–42.61)	0.93	(0.42–2.05)	0.92	(0.41–2.07)
Marital: Divorced	0.90	(0.44–1.84)	0.83	(0.46–1.50)	0.63	(0.36–1.10)
Ethnicity: Buli						
Ethnicity: Kusasi	1.10	(0.73–1.65)	1.10	(0.71–1.70)	0.88	(0.54–1.43)
Ethnicity: Frafra	1.12	(0.77–1.62)	1.17	(0.75–1.82)	1.13	(0.71–1.77)
Ethnicity: Kassem	1.68**	(1.06–2.67)	1.00	(0.66–1.50)	1.41*	(0.94–2.12)
Ethnicity: Other	1.14	(0.70–1.84)	1.40	(0.90–2.18)	1.14	(0.73–1.79)
Religion: Christian (ref)						
Religion: traditional	1.13	(0.76–1.68)	0.70**	(0.51–0.95)	0.72**	(0.52–0.99)
Religion: Muslim	0.79	(0.57–1.08)	1.15	(0.85–1.54)	1.05	(0.77–1.42)
Religion: Other	0.56	(0.26–1.19)	0.43***	(0.27–0.68)	0.43***	(0.26–0.71)
Not insured (ref)	-		-		-	
Insured	1.37**	(1.08–1.76)	1.29***	(1.09–1.52)	1.27***	(1.07–1.52)
Constant	4.20***	(1.76–10.04)	3.22***	(1.57–6.57)	4.29***	(2.11–8.71)
Observations	6,163		6,163		6,163	
Wald chi-square	171.62		244.11		271.53	
Pseudo R^2^	0.0576		0.0878		0.0882	

Notes: Table reports results from logistic regression models. ORs denotes Odds ratio from Logistic regression. 95% CI denotes 95% confidence interval. In all regressions standard errors are clustered at enumerator area level. ***, ** and * denote statistical significance at 1%, 5% and 10% respectively

Table 4 presents the effect of the CHPS + project on immunizations. Two main outcomes are used to assess the impact on immunization: (1) an indicator that a child one year or older received a measles vaccination and (2) an indicator that children over one year received three doses of DPT vaccination. The results show significant improvements in immunization coverage between baseline and endline. The likelihood of measles immunization and three doses of DPT immunization increases by 10% and 35% respectively between baseline and end line at both are statistically significant at conventional levels.

The above improvements in immunization and allied services could be attributed to the active mobilization of women in the communities to visit health facilities for ANC, PNC, and related services, as recounted by a mother captured in the following text from a focus group discussion:

***“*** *Their role in maternal and child health as volunteers as I earlier mentioned is to announce to the pregnant women and nursing mothers on the days they are to come for ANC, PNC, and CWC services. They also trace these women if they fail to turn up on such days for their respective services.* **(FGD_Men_Sheaga_Talensi)**

Other determinants of immunization coverage are the age of the mother, occupation, ethnicity, and insurance coverage. The likelihood of being immunized is increasing in the age of the mother. Also, children born to currently or previously married mothers are less likely to be immunized compared with children born to never married women. Immunization coverage is higher among children born to mothers with some occupation compared with those who have no occupation. Children born to Frafra, Kusasi, or Kassem/Nankani ethnic groups were less like to have both immunizations. Also, children whose mothers were covered by the National Health Insurance Scheme were less likely to be immunized compared with those born to mothers without health insurance.

**Table 4:** Effects of KOICA CHPS + on immunizations

	(1)		(2)	
	**Measles vaccination**		**3 doses of DPT vaccine**	
VARIABLES	ORs	95% CI	ORs	95% CI
Baseline (ref)				
End line	1.10**	(1.02–1.19)	1.35***	(1.20–1.52)
Age: 15–19 (ref)				
Age: 20–24 years	2.32***	(1.74–3.10)	1.68***	(1.16–2.45)
Age: 25–29 years	3.09***	(2.31–4.13)	1.87***	(1.27–2.76)
Age: 30–34 years	3.39***	(2.49–4.62)	2.13***	(1.37–3.33)
Age: 35–39 years	4.63***	(3.20–6.72)	2.61***	(1.64–4.15)
Age: 40–44 years	5.87***	(3.99–8.63)	3.34***	(1.84–6.06)
Age: 44–49 years	9.45***	(5.24–17.05)	3.07***	(1.59–5.93)
Education: none (ref)				
Education: primary	0.98	(0.83–1.17)	0.80	(0.61–1.05)
Education: Middle/JHS	0.94	(0.76–1.17)	1.02	(0.72–1.46)
Education: secondary+	0.83	(0.65–1.07)	0.92	(0.65–1.30)
Wealth: poorest (ref)				
Wealth: Poorer	1.08	(0.89–1.32)	1.24	(0.93–1.67)
Wealth: Middle	1.06	(0.85–1.30)	1.17	(0.86–1.59)
Wealth: Richer	1.09	(0.85–1.38)	1.08	(0.80–1.45)
Wealth: Richest	1.04	(0.83–1.30)	1.13	(0.83–1.53)
Occupation: none (ref)				
Occupation: Farming	1.17	(0.94–1.44)	1.28*	(0.96–1.69)
Occupation: Trading	1.38**	(1.07–1.78)	1.27	(0.93–1.75)
Occupation: hairdressing	1.50***	(1.15–1.96)	1.51**	(1.07–2.14)
Occupation: Student	1.53	(0.87–2.71)	1.45	(0.76–2.77)
Occupation: Other	1.08	(0.83–1.40)	1.06	(0.75–1.51)
Marital: Never married (ref)				
Marital: married	0.99	(0.75–1.31)	0.93	(0.60–1.43)
Marital: Widowed	1.18	(0.50–2.81)	0.57	(0.21–1.58)
Marital: Divorced	1.33	(0.82–2.15)	0.92	(0.48–1.75)
Ethnicity: Buli (ref)				
Ethnicity: Kusasi	0.86	(0.64–1.14)	0.67*	(0.44–1.02)
Ethnicity: Frafra	0.99	(0.75–1.30)	0.65**	(0.44–0.98)
Ethnicity: Kassem	1.20	(0.90–1.60)	0.55***	(0.38–0.79)
Ethnicity: Other	1.02	(0.73–1.43)	0.82	(0.53–1.28)
Religion: Christian (ref)				
Religion: traditional	0.90	(0.64–1.26)	1.26	(0.81–1.94)
Religion: Muslim	0.95	(0.79–1.16)	1.13	(0.86–1.49)
Mother not insured (ref)				
Mother insured	0.54***	(0.47–0.63)	0.42***	(0.33–0.52)
Constant	0.91	(0.55–1.50)	4.83***	(2.48–9.40)
Observations	5,330		5,332	
Wald chi-square	294.30		236.67	
Pseudo-R^2^	0.0487		0.0597	

Notes: Table reports results from logistic regression models. ORs denotes Odds ratio from Logistic regression. 95% CI denotes 95% confidence interval. In all regressions standard errors are clustered at enumerator area level. ***, ** and * denote statistical significance at 1%, 5% and 10% respectively

Finally, Table 5 presents the results for the effect of the project on seeking appropriate care for children with childhood symptoms. Again, two outcome indicators are considered: (1) an indicator for taking Oral Rehydration Salts (ORS) by children with diarrhea, and (2) an indicator for seeking care at health facilities for children showing symptoms of upper respiratory infections (URI). The sample for both regressions is restricted to children showing both symptoms, thus the lower sample sizes for those regressions.

The results show an increase in the likelihood of both outcomes at the end line compared with the baseline, although neither is statistically significant at the 95% level. The results also show that those children with diarrhea were 41% more likely to have had ORs at the end line compared with the baseline. Also, children with URI were 38% more likely to receive care at a health facility in the end line compared with baseline. Table 5 also shows that the age of the mother, education and ethnicity are significant determinants of children receiving ORS when they have diarrhea or seeking care for URI.

**Table 5:** Effects of the CHPS + Project on ORS use and health seeking for URI

	Took ORS when ill with diarrhea		Sought care at facility when ill with URI	
VARIABLES	ORs	95% CI	ORs	95% CI
Baseline (ref)				
End line	1.41	(0.87–2.26)	1.38	(0.82–2.32)
Age group (15–19 ref)				
Age group: 20–24	2.38*	(0.95–5.95)	2.48	(0.78–7.89)
Age group: 25–29	2.26*	(0.88–5.76)	2.60	(0.79–8.54)
Age group: 30–34	3.45**	(1.25–9.52)	1.90	(0.59–6.15)
Age group: 35–39	2.88*	(0.98–8.51)	3.55**	(1.13–11.09)
Age group: 40–44	1.65	(0.52–5.20)	10.49***	(2.06–53.45)
Age group: 45–49	6.35**	(1.23–32.70)	30.12***	(4.06–223.40)
Education: None (ref)				
Education: Primary	1.94**	(1.09–3.43)	1.09	(0.59–2.00)
Education: JHS/JSS	0.92	(0.43–1.96)	0.72	(0.31–1.65)
Education: SHS+	1.13	(0.60–2.14)	0.53**	(0.29–0.96)
Wealth: Poorest (ref)				
Wealth: poorer	0.88	(0.45–1.72)	0.80	(0.38–1.72)
Wealth: Middle	0.40***	(0.21–0.77)	0.48*	(0.23–1.00)
Wealth: Richer	0.88	(0.45–1.72)	0.58	(0.26–1.28)
Wealth: Richest	0.85	(0.43–1.66)	1.63	(0.53–4.98)
Occupation none (ref)				
Occupation: farming	1.64	(0.77–3.49)	0.69	(0.22–2.10)
Occupation: Trading	1.24	(0.47–3.24)	0.92	(0.36–2.35)
Occupation: Hairdressing	1.51	(0.63–3.62)	1.23	(0.45–3.36)
Occupation: Student	7.63**	(1.01–57.44)	1.70	(0.16–17.69)
Occupation: other	0.82	(0.32–2.12)	0.54	(0.20–1.44)
Marital: Never married (ref)				
Marital: Married	1.11	(0.60–2.06)	0.96	(0.36–2.62)
Marital: Widowed	3.94	(0.39–39.43)	0.47	(0.11–2.10)
Marital: Divorced	0.44	(0.13–1.49)	0.84	(0.13–5.22)
Ethnicity: Buli (ref)				
Ethnicity: Kusasi	1.32	(0.51–3.37)	7.76**	(1.08–56.00)
Ethnicity: Frafra	2.51**	(1.04–6.03)	8.29**	(1.34–51.30)
Ethnicity: Kassem	0.40	(0.13–1.26)	10.04**	(1.30–77.84)
Ethnicity: Other	1.13	(0.43–2.93)	5.56*	(0.81–38.35)
Religion: Christian (ref)				
Religion: traditional	0.82	(0.30–2.27)	0.99	(0.28–3.58)
Religion: Muslim	1.27	(0.73–2.23)	0.96	(0.53–1.73)
Mother not insured (ref)				
Mother is insured	1.13	(0.77–1.67)	1.11	(0.61–2.00)
Constant	0.19*	(0.04–1.00)	0.10*	(0.01–1.11)
Observations	654		495	

Notes: Table reports results from logistic regression models. ORs denotes Odds ratio from Logistic regression. 95% CI denotes 95% confidence interval. In all regressions standard errors are clustered at enumerator area level. ***, ** and * denote statistical significance at 1%, 5% and 10% respectively

### Summary, conclusions, and discussion

The KOICA CHPS + aimed to improve Ghana’s existing health delivery programme through strengthening components of the CHPS programme as originally designed in its formative years. These interventions included reactivating and empowering community actors to actively participate in health delivery, strengthening the referral systems through the provision of community transport systems, and providing medical and other equipment to CHPS + compounds, subdistrict health centres and district hospitals, to facilitate effective health delivery. In addition, skills improvement training was provided to personal at all levels, from the community to the district level, including management and leadership training to subdistrict, district and regional managers and supervisors. Reference details above.

Results show that overall, the programme has had a strong positive impact on the functioning of CHPS in the UER. This manifested in the increased likelihood that women are visited at home by CHVs and CHOs. This improvement in CHPS functioning resulted in concomitant improvement in key maternal and child health indicators. ANC coverage improved significantly as illustrated by a 76% increase in the likelihood of pregnant women having four or more ANC visits. Similarly, both the odds of delivering in a health facility or assisted in delivery by skilled health personnel increased significantly between the baseline and the endline. In addition, there was a significant improvement in immunization coverage. The results also showed improvements in the likelihood of taking ORS and seeking care at a health facility for children when they have URI. While not statistically significant, the magnitude of the change in the coefficients is worthy of note.

These improvements are significant given the context of the period when the endline survey was conducted. The endline survey was conducted during the third quarter of 2020 (June - August) which coincided with the period of intense transmission of the COVID-19 pandemic in Ghana and thus likely to negatively impact facility attendance. Indeed, evidence from other parts of the world show that the COVID-19 pandemic had a disruptive impact on facility attendance [33]. The World Health Organization (WHO) reports that analysis of “five key essential health service indicators, including outpatient consultation, inpatient admission, skilled birth attendance, treatment of confirmed malaria cases and provision of the combination pentavalent vaccine in 14 countries finds a sharp decline in these services between January and September 2020 compared with the two previous years” [34]. Similarly, work done by UNICEF Ghana also highlighted how nearly one million children below one year of age have been missing out on routine essential health services [35].

The results of this activity have institutional significance. Achieving these results demonstrates that modest financial investment and technical engagement by Ghana Health Service and other stakeholders in the health sector could result in a successful scale-up of GEHIP. The KOICA CHPS + initiative has successfully created a region of excellence for primary health care development, demonstration, and action, as envisioned by Fig. 1 and by knowledge management activities of GEHIP in the past [36, 37]. The success of this initiative attests to the need to utilize the UER as a demonstration region where other regional and district health management teams can interact with CHPS + management stakeholders, learn directly from their success, and transfer learning to other regions of Ghana. KOICA has launched a new initiative designed to develop health systems capacity in a newly created neighboring region, where poverty is extensive and health development challenges are severe. Addressing the need to develop primary care in this new initiative should now benefit from UER capacity to achieve remarkable progress. Technical support, if adequately purveyed, should include CHPS + participating DHMT and regional leadership. And, the process of exchange should include other regions of Ghana to catalyze the scale-up of learning and success that CHPS + represents.

## Data Availability

The data used in this paper are available from the corresponding author upon reasonable request.

## Abbreviations

ANC: Antenatal Care
CHOs: Community Health Officers
CHNs: Community Health Nurses
CHVs: Community Health Volunteers
CHMCs: Community Health Management Committees
CHPS: Community-based Health Planning and Services
CBPHC: Community-Based Primary Health care
DHMTs: District Health Management Teams
DHMT: District Health Management Teams
EAs: Enumeration Areas
FGDs: Focus Group Discussions
GEHIP: Ghana Essential Health Intervention Programme
IDIs: In-depth Interviews
MoH: Ministry of Health
PHC: Primary Health Care
KOICA: Korea International Cooperation Agency’s
RHMT: Regional Health Management Team
SERC: Sustainable Emergency Reference System
TBAs: Traditional Birth Attendants
UER: Upper East Region.
